# The [ ^13^C]octanoic acid breath test for gastric emptying quantification: A focus on nutrition and modeling

**DOI:** 10.1002/lipd.12352

**Published:** 2022-07-07

**Authors:** Johanna von Gerichten, Marwan H. Elnesr, Joe E. Prollins, Ishanki A. De Mel, Alan Flanagan, Jonathan D. Johnston, Barbara A. Fielding, Michael Short

**Affiliations:** ^1^ Department of Nutritional Sciences, Faculty of Health and Medical Sciences University of Surrey Guildford UK; ^2^ Department of Chemical and Process Engineering, Faculty of Engineering and Physical Sciences University of Surrey Guildford UK; ^3^ Section of Chronobiology, Faculty of Health and Medical Sciences University of Surrey Guildford UK

**Keywords:** [^13^C]octanoic acid, biophysical model, breath test, gastric emptying, mathematical model, solid food

## Abstract

Gastric emptying (GE) is the process of food being processed by the stomach and delivered to the small intestine where nutrients such as lipids are absorbed into the blood circulation. The combination of an easy and inexpensive method to measure GE such as the CO_2_ breath test using the stable isotope [^13^C]octanoic acid with semi‐mechanistic modeling could foster a wider application in nutritional studies to further understand the metabolic response to food. Here, we discuss the use of the [^13^C]octanoic acid breath test to label the solid phase of a meal, and the factors that influence GE to support mechanistic studies. Furthermore, we give an overview of existing mathematical models for the interpretation of the breath test data and how much nutritional studies could benefit from a physiological based pharmacokinetic model approach.

## INTRODUCTION

Gastric emptying (GE), in simple terms, is the delivery of food from the stomach into the small intestine. An appropriate passage of micro‐ and macro‐nutrients as well as indigestible particles through the gastrointestinal tract is only possible with a regulated GE process, allowing the absorption of digested food into the blood (Liu et al., 2020). Today, measuring GE aids the diagnosis and treatment of gastrointestinal‐related disorders such as gastroparesis (Krishnasamy & Abell, 2018), type 2 diabetes (Linnebjerg et al., 2008), and dyspepsia‐related disorders such as Crohn's disease and Inflammatory Bowel disease (Nóbrega et al., 2012). Accurate GE tests allow for GE rate modifying drugs, which adjust the GE rate of the individual affected, to be tested and assessed (Maurer, 2012; Odunsi et al., 2009).

In nutrition, GE also plays an important role, since a slower rate of GE would lead to a lower postprandial rise in macronutrients, and consequently a lower postprandial burden on the clearance of these macronutrients from the circulation. This is particularly important for postprandial plasma triacylglycerol concentrations, with impaired clearance being related to inflammation and cardiovascular disease (Bickerton et al., 2007; Wallace et al., 2010). The attenuation of the postprandial increase in blood glucose has long been a goal for improved metabolic health. As reviewed elsewhere, small differences in GE can have a major impact on postprandial glycaemia in health and type 2 diabetes (Mihai et al., 2018). Moreover, through neural pathways, GE is linked to satiety (Clegg & Shafat, 2009), appetite and hunger and their hedonic aspects, as well as to chronic food intake and energy homeostasis (Goyal et al., 2019).

Several different techniques such as ultrasound, electrogastrography (EEG), magnetic resonance imaging (MRI), double sampling intubation (Beckers et al., 1988) and Wireless Sports Capsule Technology (WMC) have been used to measure GE (Bruno et al., 2013; Liu et al., 2020). In a clinical environment, GE has traditionally been measured using radioscintigraphy, which is regarded as the gold standard (Abell et al., 2008). The disadvantage of this method is the use of the radionuclide Technetium‐99 in children and pregnant woman, expensive scanning equipment and the exclusion of patients with motility‐related issues, for example, elderly persons and people with Parkinson's disease (Jackson & Bluck, 2005). Another direct technique for the measurement of gastric emptying is MRI, which shows excellent correlations with the scintigraphy method (Feinle et al., 1999). Although there is no need for radiolabelled tracers with MRI, the method still requires the availability of complex equipment and trained personnel.

An alternative to direct imaging techniques is the [^13^C]octanoic acid breath test ([^13^C]‐OABT), which measures the GE of solid food, developed by Ghoos et al. (1993) and which, as it is based on a stable isotope technique, is harmless and can be safely used in children and pregnant women.

To measure GE of the liquid phase of a meal, the ^13^C label is incorporated into acetate (see later). This may be more suitable for exercise studies as evaluated by Van Nieuwenhoven et al. (1999). The [^13^C]‐OABT involves eating a test meal containing the stable isotope and collecting serial breath samples, for example, using a straw and glass tube, which can be done without the need for specialist equipment. The test has been validated against scintigraphy as well as MRI (Camps et al., 2018; Ghoos et al., 1993). Underlying the simplicity of this description, however, are pitfalls and assumptions that require scrutiny to enable accurate use of the test.

Octanoic acid (caprylic acid) is a medium chain fatty acid used for gastric emptying because of its rapid turnover in the liver without incorporation into chylomicrons (in contrast to longer chain fatty acids). However, octanoic acid is oxidized in the liver following clearance from the circulation as CO_2_, also representing in vivo kinetics of fat absorption and β‐oxidation (Kim et al., 2016). Fat malabsorption and altered capacity to oxidize fatty acids from the diet are linked to various metabolic disorders such as cardiac ischemia, failing heart, and cancer (Adeva‐Andany et al., 2019; Harjes et al., 2016; Jaswal et al., 2011). There are not many examples of research using the [^13^C]‐OABT in common dietary food oils or actual meals and therefore our knowledge of the effect of lipids in mixed meals on gastric emptying and their bio‐accessibility from solid food for human metabolism is limited (Giezenaar, Lange, et al., 2018; Hoad et al., 2011; Robertson et al., 2002; Somaratne et al., 2020). Stable isotope‐labeled triglycerides have also been used as substrates in breath tests as a marker for pancreatic function, as pancreatic lipase is mainly responsible for intestinal lipolysis (Keller et al., 2014; Vantrappen et al., 1989). This review, however, focusses in detail on the [^13^C]‐OABT, its practicalities and the mathematic modeling, to showcase fatty acid—based breath tests and their possibilities as non‐invasive tests for nutritional studies related to fat malabsorption, liver function, digestion, and gastric emptying.

Recent reviews on the use of the [^13^C]‐OABT have covered the basic principles (Keller et al., 2021; Liu et al., 2020), physiology (Goyal et al., 2019), diagnostic application (Krishnasamy & Abell, 2018) and refinement of the equations used to derive GE parameters (Sanaka & Nakada, 2010). The aim of this review is to provide a critical overview of the test and practical considerations for planning its use in the context of nutrition studies. We will give a collection of the basic equations used for the data processing and review mathematical and statistical techniques used to interpret GE results, followed by a review of semi‐mechanistic pharmacokinetic modeling of GE rate using the [^13^C]‐OABT. To the best of the authors' knowledge, this is the first review that focuses on GE mathematical modeling, reviewing both empirical and semi‐mechanistic approaches and how these can be used to better understand and predict GE in nutrition studies.

## OVERVIEW OF THE [ ^13^C]OCTANOIC ACID BREATH TEST TO MEASURE GASTRIC EMPTYING

### Study requirements for [ ^13^C]‐OABT

It is important to adhere to validated study protocols as the [^13^C]‐OABT is dependent on a variety of digestive and metabolic processes. Therefore, participants should fast for at least 10 h before the test and should avoid food with a high natural amount of [^13^C] such as corn, broccoli, sugarcane, and pineapple (most tropical fruit) for 48 h. Furthermore, drugs that can affect gastric motor function, for example, smooth muscle relaxants, should be withdrawn from 48 to 72 h before the test, as well as alcohol. The [^13^C]‐OABT can also be influenced by physiological, and demographic factors such as age (Bonner et al., 2015), sex (Kim & Kim, 2020), weight (Cardoso et al., 2007; Jackson, Leahy, et al., 2004), exercise (Davis et al., 2020; Lang Lehrskov et al., 2018), feedback of intestinal hormones (Moran et al., 2001), and even the amount of [^13^C]octanoic acid (as recently reviewed in Liu et al. (2020) and Keller et al. (2021)).

The derivation of GE parameters requires an estimation of carbon dioxide output, based on body surface area (see later section). Therefore, prior to the test, at least the weight and height of each participant must be determined. Ghoos et al. (1993) proposed using a height‐weight formula developed by Haycock et al. (1978) to calculate surface area empirically:

(1) $$\mathrm{SA}=W^{0.5378}\;H^{0.3964}\times 0.24625$$

where, *SA* is the body surface area in m^2^, *W* is weight in kg and *H* height in cm.

### Test meal containing [ ^13^C]octanoic acid

The meal widely used for the [^13^C]‐OABT has been a mixed meal composed of scrambled egg, a fat spread, and toast based on the standardized meal protocol of Ghoos et al. (1993), Abell et al. (2008) Donohoe et al. (2009) and European guidelines for clinical applications discussed in the recent report of an expert panel (Keller et al., 2021). The [^13^C]octanoic acid, between 100 and 200 mg (Keller et al., 2021), is mixed with raw egg yolk before cooking. This allows the octanoic acid to be representative of the solid phase of the meal (Ghoos et al., 1993). A further test meal has been proposed in which the [^13^C]octanoic acid is mixed with egg yolk before incorporating into a muffin and was used in a commercially available [^13^C]‐OABT kit (Perri et al., 2010) which was proposed to be used in a clinical setting. The muffin meal in [^13^C]‐OABT has been validated against scintigraphy with a good correlation for half‐times (t½ correlation *r* = 0.8–0.9) (Bromer et al., 2002; Chey et al., 2001). The calorific content (350 kcal) of the muffin as well as the time of sample collection (minimum 4 h) was tested against electrogastrography with only moderate correlations for the half‐time (t½ correlation *r* = 0.67) (Gonlachanvit et al., 2001). However, the muffin was also compared against the standard meal of egg on buttered toast by [^13^C]‐OABT with good agreement of half‐time (Perri et al., 2010).

### Gastric emptying—physiology of the stomach, mechanism of solid food with tracer and tracer metabolism

The process of GE has been well‐summarized elsewhere (Goyal et al., 2019; Liu et al., 2020) and is briefly described in Table 1 in the context of the suitability of a tracer to calculate the in vivo rate. Octanoic acid is a medium chain fatty acid which is appropriate to be used in the breath test because of its rapid delivery into the portal blood, bypassing the formation into chylomicrons, as mentioned above. The octanoic acid used for the [^13^C]‐OABT has one carbon atom labeled with the stable isotope ^13^C instead of the natural ^12^C, allowing to track [^13^C]CO_2_ as a specific marker of the given octanoic acid oxidation. However, it cannot be used to directly reflect the postprandial metabolism of longer chain fatty acids in food, including oxidation, for which [^13^C]palmitic acid, for example, can be used (Roberts et al., 2008).

**TABLE 1:** Process description in steps of [^13^C]octanoic acid as a tracer of gastric emptying

Process	Physiological process	[13C]octanoic acid tracer metabolism
Filling phase	Food is ingested and fills the first part of the stomach, the fundus, and proximal part of the corpus. The upper stomach, including fundus, relaxes to store food.	[^13^C]octanoic acid is incorporated into the solid phase of a test meal and enters the stomach.
Early pumping phase (Tension contraction)	The proximal stomach starts slow contractions allowing the fast emptying of liquids, as well as the solid food to be mixed and broken down with gastric acid and pepsin to form chyme. Small enough food particles gradually move into the pylorus.	The lag‐phase reflects the time until the [^13^C]octanoic acid movement into the small intestine reaches maximal level and is described by the lag‐time (t_lag_), the time [^13^C]CO_2_ excretion is at maximal level.
Late pumping phase (peristalsis)	Solid food is transferred to the pylorus by wave‐like contractions that gradually strengthen and at the same time partially pushed back by the pylorus to ensure grinding and mixing. The antrum as part of the pylorus must fill to a certain level before food is moved through a muscular valve to the small intestine.	The linear emptying phase is described by the time taken for 50% of the [^13^C]octanoic acid to be emptied into the small intestine, the gastric half‐emptying time (t_1/2_), which is described by the time when half [^13^C]CO_2_ is excreted.
Absorption, liver metabolism and breath exhalation	[^13^C]octanoic acid is absorbed rapidly in the small intestine and enters the hepatic portal vein. Once taken up by the liver, the fatty acid undergoes β‐oxidation, and exits the liver as carbon dioxide dissolved in the bicarbonate pool. In the lungs it is excreted from the body as breath.	Appearance of [^13^C] in breath carbon dioxide and loss through circulation and metabolism. The rate of exhalation is used to calculate the rate of GE.

Upon ingestion of the test meal containing [^13^C]‐labeled octanoic acid and following GE, the labeled fatty acid is rapidly absorbed in the small intestine and transported to the liver in the hepatic portal vein (Figure 3), where it is oxidized into CO_2_ and enters as such into the circulation. The general hypothesis for the [^13^C]‐OABT regarding the human physiology, is that the rate limiting step for the CO_2_ release is the delivery from stomach to small intestine, the rate of GE.

However, in light of more physiological modeling approaches further steps of octanoic acid metabolism need to be considered (Figure 3). The [^13^C]‐OABT is essentially a one‐bolus test where participants ingest a single defined dose of [^13^C]octanoic acid and [^13^C]CO_2_ results are calculated as percent dose recovered. One consideration is that not 100% of the [^13^C]octanoic acid dose is absorbed by the small intestine, but directly excreted as shown for other fatty acids (Jones et al., 1999). Entering the liver, a two‐step process leads to the degradation of octanoic acid with [^13^C]CO_2_ as a product. The first step is the β‐oxidation of octanoic acid which results in [^13^C]acetyl‐CoA. A set of (fast) metabolic losses might occur after this first step as acetyl‐CoA can be used for cholesterol synthesis (and therefore bile salts and steroid hormones), Ketogenesis, Gluconeogenesis and *de‐novo* fatty acid synthesis (Hasenour et al., 2020; Ontko, 1972). In the second step, [^13^C]acetyl‐CoA enters the TCA cycle where it is processed into [^13^C]CO_2_ (Xiong, 2018). CO_2_ dissolved in water is in equilibrium with carbonic acid, bicarbonate, carbonate, and gaseous CO_2_ at air water interfaces such as the lung. Consequently, [^13^C]CO_2_ can be exhaled via the pulmonary circulation but is still in equilibrium with the non‐pulmonary circulation in blood and tissues (Figure 3). Metabolic recycling of CO_2_ back into the TCA cycle via pyruvate or entering the urea cycle can occur as well as entering other turnover pools such as integration into bone, urine and sweat (Yang et al., 2005). As a result only around half of the [^13^C]octanoic acid is exhaled as [^13^C]CO_2_ (Sanaka & Nakada, 2010).

### Data collection using breath

Data analysis of expired [^13^C]CO_2_ requires a sufficient number of data points to be collected. Breath sampling over 4 h with samples being taken every 15 min is required for analysis by non‐linear regression (NLR) (Ghoos et al., 1993). A simplified method that only samples at six time points for the same 4 h period and that uses a generalized linear regression method (GLR) was developed later (Lee, 2000; Szarka et al., 2008). The within‐subject variability in healthy volunteers was evaluated using the muffin meal and breath samples every 15 min over 4 h to be around 17% for the t½ (Perri et al., 2010). The inter‐individual variability was reported to be 27% for t½ using the egg‐fat‐toast standard meal with healthy volunteers (Peracchi et al., 2000). Alternatively, sampling every 15 min for 6 h has been advocated in a research setting (Clegg & Shafat, 2010).

Breath analysis can be measured either with isotope‐ratio mass spectrometry (IRMS) or infrared spectrometry to differentiate between [^13^C]CO_2_ and [^12^C]CO_2_ content in the breath (Keller et al., 2021). Commercially available and relatively cheap breath analysers based on infrared techniques with standardized protocols are available (e.g., isotope selective non‐dispersive infrared spectroscopy [IRIS] Kibion®Dynamic, Kibion GmbH Bremen, Germany).

An overview of the [^13^C]‐OABT methodology modified from Bruno et al. (2013) is shown in Table 2.

**TABLE 2:** Methodology for carrying out [^13^C]‐OABT (based on Bruno et al., 2013)

Step 1	Participants should fast at least 10 h before the [^13^C]‐OABT and adhere to the 48 h exclusion protocol of certain food, drugs etc.
Step 2	Weight and height are determined for each patient to calculate body surface area.
Step 3	Breath samples are collected for each patient before meal is consumed (*t* = 0).
Step 4	[^13^C]‐labeled meal should be consumed in 10 min. Some water is provided with the meal.
Step 5	Breath samples are collected in 15 min intervals over 4 h. Participants ideally stay seated for the sampling period but should avoid the following after consuming the meal during the study: eat, smoke, sleep and anything more than light exercise.
Step 6	Breath sample analysis using Isotope Ratio Mass Spectrometry (IRMS) or Infrared Spectrometry (IR) to distinguish [^13^C]CO_2_ from [^12^C]CO_2_ in exhaled breath.
Step 7	Data interpretation using mathematical modeling.

## FACTORS AFFECTING GASTRIC EMPTYING

The standard [^13^C]‐OABT is based on the [^13^C]octanoic acid bound to a solid meal and does not represent the liquid phase. The liquid in a mixed meal empties faster and can be traced using [^13^C]sodium acetate (Braden et al., 1995). Labelling of the solid phase is achieved by incorporating the [^13^C]octanoic acid into lipophilic egg yolk, which has been shown to result in 96% retention after 3 h in a “peptic milieu” (Ghoos et al., 1993). However, Gonlachanvit et al. has challenged this by obtaining comparable parameter values using [^13^C]sodium octanoate dissolved in water and a dry muffin mix baked in a microwave without egg (Gonlachanvit et al., 2001). The following section highlights factors affecting GE that should be considered when designing nutritional studies, from previous research that did not necessarily use the [^13^C]‐OABT.

### Nutritional studies

Increasing the meal size has been shown to delay GE due to energy content, volume, or both effects (Gonlachanvit et al., 2001; Jackson, Bluck, et al., 2004; Peracchi et al., 2000) (Table 3). Although the common consensus seems that a test meal should exceed 300 kcal in a clinical context (Keller et al., 2021), it is not clear if a high‐energy meal increases the inter‐individual variability of the [^13^C]‐OABT.

**TABLE 3:** Impact of different meals on measured gastric emptying ([^13^C]‐OABT) parameters

	Peracchi et al. (2000)		Gonlachanvit et al. (2001)		Jackson, Bluck, et al. (2004)		
Study size	*n* = 10		*n* = 14		*n* = 12		
Meal type	Egg on toast		Muffin (no egg)		Egg on toast		
Meal calories (kcal)	250	550	250	350	238	476	714
Carbohydrate	42%	45%	45 g	63 g	45%		
Fat	40%	35%	5.4 g	7.5 g	40%		
Protein	18%	20%	5.7 g	8.0 g	15%		
Data collection	every 15 min for 5 h		every 15 min for 6 h		every 15 min for 4 h, then every 30 min for 2 h		
t½ (min)	156^ª^ (112–256)	289^ª^ (162–346)	153 ± 7^*b*^	177 ± 7^*b*^	202^*c*^	255 ± 9 b	274^*c*^
t_lag_ (min)	111^ª^ (82–168)	175^ª^ (104–455)	95 ± 5^*b*^	114 ± 4^*b*^	152^*c*^	190 ± 8 b	196^*c*^

*Note*: The three studies included here were randomized cross‐over studies; the article by Gonlachanvit et al. (2001) used [^13^C]sodium octanoate.

^ª^ Median with range in ().

^*b*^ Mean ± sem.

^*c*^ Calculated from reported mean differences in original article.

Gastric emptying of solids and liquid food differs significantly. For example, a study where participants were given an isocaloric and isovolumetric meal of whole apples, apple puree and apple juice with water resulted in faster GE of puree and juice compared to whole apple as determined by MRI (Krishnasamy et al., 2020).

Food viscosity as well as food material properties (physicochemical) such as composition and structure have an impact on GE, for example, a higher viscosity keeps food longer in the digestive system and larger/harder food particles take longer to be disintegrated as reviewed in Somaratne et al. (2020). The chemical properties can also play a role as demonstrated in a study comparing different types of fatty acids and concluding that omega‐3 fatty acids increase gastric emptying rate compared to omega‐6, monounsaturated and saturated fatty acids (Robertson et al., 2002). Other studies showed that a higher fat content in the diet accelerated GE in the long term (Clegg et al., 2011); whereas it is well‐established that fat in a one‐off single meal delays GE as reviewed by the same researchers (Clegg & Shafat, 2009), whilst a higher content of fiber (Benini et al., 1995) and protein (Giezenaar, Lange, et al., 2018) in food delayed GE. A recent study investigated the effect of slowly digestible carbohydrates on GE but the [^13^C]octanoic acid was delivered by mixing into each yogurt‐based test meal. The assumption was made that the protein and fat contents of the yogurt allowed the [^13^C]octanoic acid to be readily soluble and evenly distributed in the semi‐solid matrix. This assumption did not appear to have been tested (Chegeni et al., 2022).

### Age and sex

Faster GE rates observed in younger compared to older children might be confounded by differences in diet (Bonner et al., 2015). However, a study in children aged 6–18 years, reported delayed GE in younger children as well as delayed GE in females (Orsagh‐Yentis et al., 2021). The study used a recently developed breath test using [^13^C]spirulina in the egg instead of [^13^C]octanoic acid which had previously been approved by the Food and Drug Administration for adults in the United States. In healthy adults (mean age 50 years), (Hellmig et al., 2006) no significant impact of sex, age and body mass index (BMI) on GE with the standard test meal of scrambled egg and toast was found. In contrast, healthy female adults in a small study (*n* = 8; average age of 23 years) that were BMI matched and given a protein drink showed a slower gastric emptying than male participants (Giezenaar, Luscombe‐Marsh, et al., 2018). The same group compared the young male adults with older male adults (average 75 years) and determined no difference in GE between the age groups (Giezenaar et al., 2020). However, GE has been shown to change across the menstrual cycle (Campolier et al., 2016).

Age as well as sex difference in GE are better defined in diseases such as dyspepsia, where premenopausal women show slower GE rates than men and postmenopausal women (Gomez Cifuentes et al., 2019; Kim & Kim, 2020).

### Exercise

A meta‐analysis from 2015 using data from 20 studies, mainly using a liquid bolus delivered pre‐exercise to measure GE, concluded that chronic low intensity exercise results in faster GE whilst high intensity exercise results in slower GE (Horner et al., 2015). However, an intervention study with young obese men could not detect a difference in GE in response to a 4 week period of regular chronic low intensity exercise (Horner et al., 2021). Similarly, healthy un‐trained adult males showed no difference in GE in a study comparing no exercise with acute 60 min low and high lower‐body exercise before breakfast (Mattin et al., 2018). In contrast, a recent study assessed physical activity with a questionnaire in 270 obese individuals and found a significantly faster GE in physically active participants for the liquid and solid phases of the test meal (Davis et al., 2020).

### Sleep and daily variation

There is evidence of daily variation in the rate of GE, although the data are limited. In the first article to demonstrate circadian variation in gastric emptying Goo et al. (Goo et al., 1987) showed that, compared to the same test meal consumed at 08.00 h, the GE half‐time was 32.3 min later following the meal consumed at 20.00 h. A more recent study, albeit in only 2 participants [1 male, 1 female], also suggested a 220% delayed GE half‐time comparing the same meal at 08.00 h to 23 h, while also suggesting sex differences with significantly more delay in the female participant (Grammaticos et al., 2015). Thus, there does appear to be evidence of time‐of‐day influences on gastric emptying rate, however, the evidence to date is sparse.

## [ ^13^C]‐OABT DATA ANALYSIS AND INTERPRETATION

### Model based on Ghoos et al. (1993)

The first model used to interpret [^13^C]‐OABT data is the method presented by Ghoos et al. (Ghoos et al., 1993) which is based on the assumption that once the solid phase of the test meal reaches the small intestine, the given [^13^C]octanoic acid is rapidly degraded into [^13^C]CO_2_. Two equations have been derived from the empirical fact that the curve of cumulative percent recovery of [^13^C]CO_2_ over time is inversely analogous to the scintigraphic curve of gastric emptying. Nowadays, most published literature on the [^13^C]‐OABT use the same equations with different notation or offer only minor modifications.

The first value is breath [^13^C]CO_2_ excretion rate expressed as percent dose recovered (PDR) per hour (*h*) of the given [^13^C] dose (PDR/h), calculated by Equation (2). VCO_2_ (volume of CO_2_ production) is calculated with an assumed CO_2_ production of 300 mmol/m^2^ of body surface per hour and body surface area was calculated based on (Haycock et al., 1978). APE is atom percent excreted and determined by for example, stable‐isotope ratio mass spectrometry of the [^13^C]O_2_ in the breath. APE represents the atom percentage above baseline, which means the enrichment at baseline is zero, and is therefore independent of the analytical instrument. The excretion rate is fitted by Equation (3).

(2) PDR/h=APE×VCO2hC13dose

(3) $$y_{1}=at^{b}e^{-\mathrm{ct}}$$

where, *y*_1_ is the rate of [^13^C] excreted in the breath per hour, *t* is time in hours and *a*, *b*, and *c* are parameters to be estimated from the curve‐fitting.

The second value is the percentage of total [^13^C]CO_2_ excreted in the breath of the total [^13^C] dose over time in hours where samples were taken, the cumulative PDR/h (cPDR), which is fitted by Equation (4). The cPDR is derived by the sum of areas under the curve at each given time point over the whole time period samples were taken.

(4) $$y_{2}=m{\left(1-e^{-\mathrm{kt}}\right)}^{\beta }$$

where, *y*_2_ is the percentage of cumulative [^13^C] in the breath, *t* is the time in hours, *k*, *β*, and *m* are constants, with m being the total cumulative [^13^C] recovery when time is infinite. Whereby, in a later publication Maes et al. (1994) used the first derivative of *y*_2_, which is shown as y_3_ in Equation (5).

(5) $$y_{3}=\mathrm{mk}\beta e^{-\mathrm{kt}}{\left(1-e^{-\mathrm{kt}}\right)}^{\beta -1}$$

Conducting nonlinear regression analysis allows three parameters to be derived: half‐emptying time (*t*_1/2,*b*_), the lag phase ($t_{\mathrm{la}g,b})$, and the gastric emptying coefficient (GEC), these parameters are represented by Equations ((6), (7), (8)), respectively (also see Figure 2).

(6) $$t_{\left(1/2,b\right)}=\left[-\frac{1}{k}\right]\times \ln\left[1-2^{-\frac{1}{\beta }}\right]$$

(7) $$t_{\mathrm{lag}{,}_{b}}=\frac{\ln\left(\beta \right)}{k}$$

(8) $$\mathrm{GEC}=\ln\left(a\right)$$

**FIGURE 1:**
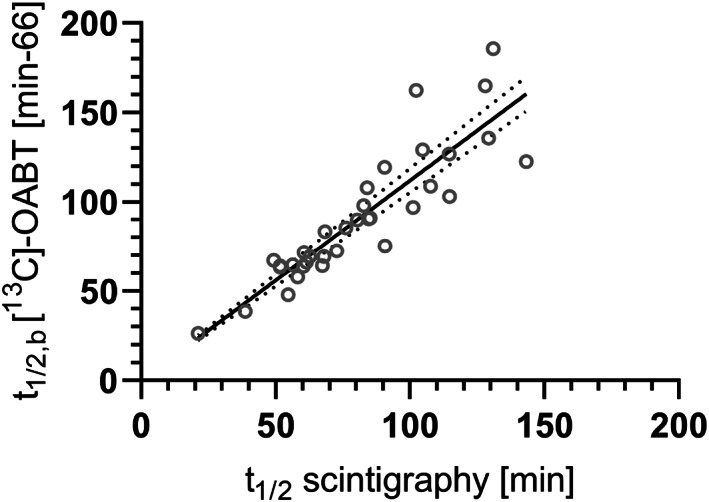
Validation of the [^13^C]‐OABT with scintigraphy. Comparison of half‐emptying times for [^13^C]‐OABT on the *y*‐axis (t_1/2,b_ which is corrected by 66 min) and scintigraphy (t_1/2_) on the *x*‐axis. [Figure shows a schematic re‐drawing based on Ghoos et al., 1993 Figure 3 B]

Ghoos et al. (1993) and Maes et al. (1994) showed a 66 min delay for the results of the [^13^C]‐OABT compared to the scintigraphic gastric emptying, and that the [^13^C]‐OABT is as reliable as the gold standard scintigraphy with parameter correlations of *r* = 0.89 for the half emptying time and *r* = 0.92 for the lag time (*n* = 42 participants, Figure 1 re‐drawn from Ghoos et al.).

The range for the parameter values determined were GEC = 2.7–4.0 ± 11%, *t*_1/2,b_ = 20–118 ± 28% and *t*_lag_ = 0–77 ± 45%. Schommartz et al. (1998) used the findings of Ghoos et al. (1993) and adjusted the equations to the scintigraphic data for half‐time and lag time as seen in Equations (8) and (9).

(9) $$t_{\left(1/2,b\right)}=\left[60\left(-\frac{1}{k}\right)\times \ln\left(1-2^{-\frac{1}{\beta }}\right)-66\right]/1.12$$

(10) $$t_{\mathrm{lag},b}=\left[\left(60\times \frac{\ln\left(\beta \right)}{k}\right)-60\right]/0.94$$

**FIGURE 2:**
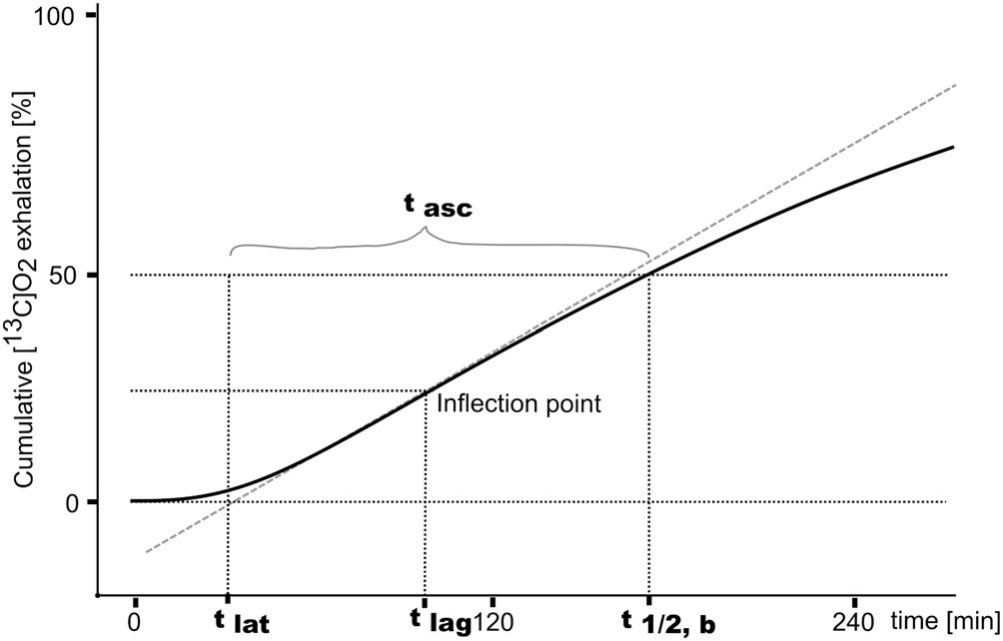
Parameters of gastric emptying by [^13^C]‐OABT. The parameters half‐time (*t*_1/2,*b*_), time to maximum excretion (*t*_lag_), the initial delay until [^13^C]CO_2_ exhalation (latency; t_lat_) and the span from latency to half‐time (t_asc_) depicted in a schematic drawing (based on unpublished data).

The same publication (Schommartz et al., 1998) pointed out that the two parameters, half‐time and lag phase, are correlated as they share estimable parameters and therefore can largely replace each other as result. Two new and uncorrelated parameters called latency time (*t*_lat_; Equation (11)) and ascension time (*t*_asc_; Equation (12)) were proposed instead, which describe the delay of the cumulative exhalation curve and the time interval from delay to half‐time, respectively (Figure 2).

(11) $$t_{\mathrm{lat}}=\frac{1}{k}\times \left(\frac{\ln\left(\beta \right)+1}{\beta -1}\right)$$

(12) tasc=−1k×ln1−2−1β+lnβ+1β−1

Although there is some signs of correlation between *t*_lat_ and *t*_asc_, the correlation is fairly weak due to variation in the results.

The parameters so far introduced are summarized in Figure 2.

### Limitations of the Ghoos et al. (1993) model

Although the work done by Ghoos et al. (1993) was regarded as a breakthrough in [^13^C]‐OABT development and various authors have relied on this work, there are several limitations with their GE model. One is that it was developed in order to derive GE parameters obtained from [^13^C]‐OABT that are suitable to be compared with scintigraphy results. Maes et al. (1994) proposed the use of a correction factor of “−66 min” to correct the [^13^C]‐OABT gastric emptying time (*t*_1/2,b_) so that it is in line with values obtained by scintigraphy. The results between [^13^C]‐OABT and scintigraphy without correction are distinctly different. Others later showed that using the correction factor can lead to negative lag phase values, and that the lag time is not only caloric content‐dependent, but also dependent on many factors like individual patient, meal volume and gravity (González et al., 2000; Schommartz et al., 1998). As a result, the use of the correction factor may increase the variability of the data set. Another study, González et al. (2000) later proposed a rate of emptying which is described as the half‐time subtracted by the lag time and therefore circumvents the use of an absorption time correction. The recently published European Guidelines allow for reporting of un‐corrected values for the [^13^C]‐OABT (Keller et al., 2021).

Another limitation of the [^13^C]‐OABT model by Ghoos et al. (1993) is the very simplified assumption that the rate of emptying solid food in the stomach is equated to the rate of CO_2_ exhaled in the breath from oxidized [^13^C]octanoic acid and in this process describing a physiological process depending on many factors with a non‐linear curve‐fitting. The assumption that the absorption time of octanoic acid from the small intestine, via the liver into the lung is so small that it can be dismissed also ignores the fact that the breath is not the only route of elimination or the possibility that some of the octanoic acid can be retained via the systemic circulation. The research group around Sanaka et al. (2005) pointed out that the accuracy of the [^13^C]‐OABT is highly dependent on the goodness‐of‐fit reached by the non‐linear curve fit, which can be quite poor considering the small number of data points and possible interaction between parameters. Another research group (Perri et al., 2005) state that the rate of CO_2_ exhalation and CO_2_ appearing in the breath is unaffected by other metabolic steps on account of studies done by Ghoos et al. (1993) and that the assumption of [^13^C]CO_2_ almost immediately appearing in breath is valid. However, discrepancies between the [^13^C]‐OABT and scintigraphy experimental results invalidates this statement and highlights the key disadvantage of [^13^C]‐OABT where it does not consider other processes contributing to GE rate. Examples of such processes are metabolic absorption and distribution and elimination by means other than exhalation of breath (Sanaka et al., 2008; Sanaka & Nakada, 2010).

### Expansion of the Ghoos et al. (1993) model

Although there has been little deviation from the model developed by Ghoos et al. (1993) some efforts have been made to expand the model to develop understanding of the GE process and introduce new parameters and methodology.

Sanaka et al. (2005) introduced the Wagner–Nelson method (Wagner & Nelson, 1964) which was originally developed for pharmacokinetic (PK) determination of orally applied drug absorption rates that are cleared via the urine. This one‐compartment model uses first‐order kinetics to reduce postabsorptive effects on [^13^C]CO_2,_ assuming that the amount of [^13^C]octanoic acid absorbed into the body is the sum of [^13^C]octanoic acid residing in the body and the amount of [^13^C]CO_2_ eliminated via breath. The rate of elimination via the breath is proportional to the amount of [^13^C]octanoic acid in the body, and still assuming that the absorption of [^13^C]octanoic acid from the small intestine to the liver is fast, the absorption rate equals gastric emptying. Results are expressed and compared in a gastric emptying flow curve which describes the fraction of [^13^C]octanoic acid remaining to be absorbed (Wagner–Nelson) and that of [^13^C]CO_2_ remaining to be exhaled (conventional [^13^C]‐OABT) over time, the adjusted calculations are shown in Equations (13) and (14).

(13) $$F_{\text{Wagner}-\text{Nelson}}\left(t\right)=1-\left[\frac{A_{\text{breath}}\left(t\right)+C\left(t\right)}{0.65}\right]/A_{\text{breath}}\left(\infty \right)$$

(14) $$F_{\mathrm{OBT}}\left(t\right)=1-\frac{A_{\text{breath}}\left(t\right)}{A_{\text{breath}}\left(\infty \right)}$$

where *A*_breath_(*t*) is the cumulative amount of [^13^C]CO_2_ excreted at time point *t* and *A*_breath_(∞) is the ultimate amount of [^13^C]CO_2_ excreted, which practically is described as the cumulative amount of [^13^C]CO_2_ at the last time point samples were taken. Finally, *C*(*t*) is the [^13^C]CO_2_ excretion rate at each time point and the constant for total elimination of [^13^C]CO_2_ is expressed as the mean value 0.65 (Sanaka et al., 2004). Past research (Perri et al., 2005) considered the clinic‐pharmacological applications of the [^13^C]‐OABT model developed by Ghoos et al. (1993) and how the model could be developed into a PK model. By implementing PK features, the model could be used to diagnose precise dosages of gastric‐modifying drugs to combat the inhibition/acceleration by gastric conditions. Perri et al. (2005) only briefly discussed this mechanism, but it was important to the overall model development as PK modeling was utilized by semi‐mechanistic models developed in future literature (Ogungbenro & Aarons, 2011). Sanaka et al. (2008) studied the detailed kinetics involved in GE of [^13^C]octanoic acid once ingested in a test meal, which was important for the validation of the discrepancy in the results of the model by Ghoos et al. (1993).

Further research (Markey & Shafat, 2013) investigated different mechanisms to determine the CO_2_ production rate (VCO_2_) from the breath and the subsequent impact on GE results. They developed new parameters for the study with VCO_2DM_ the direct measurement of rate, VCO_2PR_ the predicted rate from resting and VCO_2BSA_ the predicted rate from body surface area. Although, the study showed great potential in further explaining the [^13^C]‐OABT mechanisms, the results were not promising due to limited correlations. The assumptions developed to calculate VCO_2PR_ and VCO_2BSA_ proved inaccurate and assumed GE responses for [^13^C]‐OABT are primarily based on exhalation of breath. In the context of GE modeling, it would be interesting to explore estimations of VCO_2_, which is related to resting energy expenditure, based on variables such as lean body mass, or measured by indirect calorimetry (Wang et al., 2001).

Although the Ghoos model has been shown to yield relatively accurate results, the approach fails to explain the physiological meaning of GE parameters and the significance of these variables to GE rate. This led to research into application of semi‐mechanistic PK‐inspired modeling to analyze the identifiability of different parameters in the GE model and to identify and reasonably explain outliers in patient data.

## THE EVIDENCE FOR PHARMACOKINETIC‐BASED MODELING OF [ ^13^C]OCTANOIC ACID BREATH TEST

### Key parameters for physiological modeling

The details of a physiologically based model as well as a more concise overview are given in the publications from Sanaka et al. (2008) and Sanaka and Nakada (2010), which define the key kinetics of [^13^C]‐OABT based on four key processes. The first key process, ‘fixation’, is defined as the temporary storage of [^13^C]CO_2_ in the body as bicarbonate or metabolic product pools. This is described as having a slow turnover. The second key process, ‘retention’, is similar to fixation as it is also defined as temporary storage in body pools, however, it is described with a rapid turnover. ‘Turnover’ itself is the third key process describing the [^13^C]CO_2_ excreted that was stored in body pools and re‐entered circulation. The fourth and final key process, “loss,” is the (irreversible) elimination of [^13^C] from the body by pulmonary circulation (elimination by breath).

**FIGURE 3:**
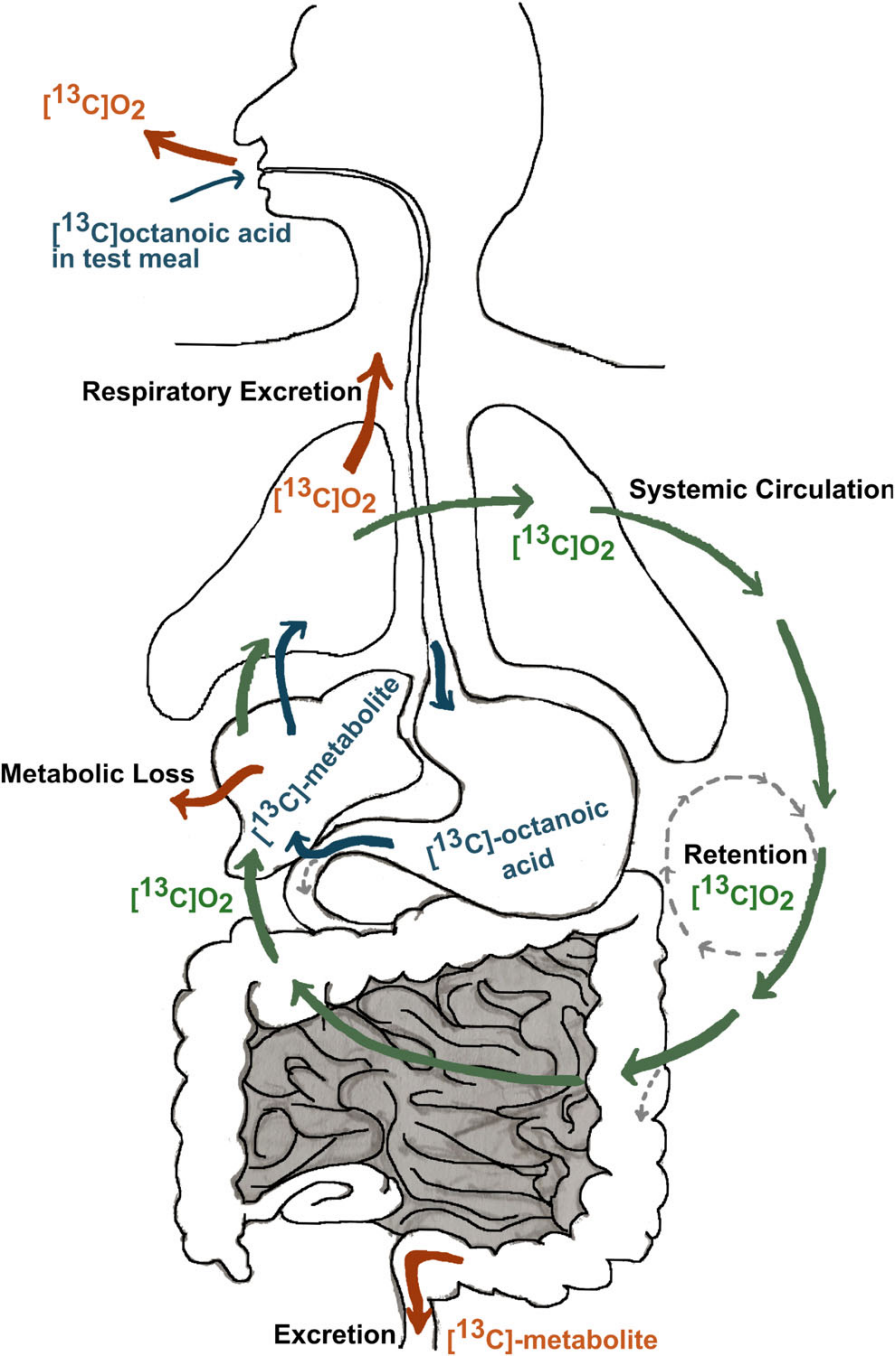
[^13^C]‐octanoic acid metabolism and physiological key parameters. (1) Distribution of [^13^C]CO_2_ into metabolic/bicarbonate pools with rapid turnover (systemic circulation)—retention. (2) Distribution of [^13^C]CO_2_ into metabolic/bicarbonate pools with slow turnover (metabolic loss)—fixation. (3) Elimination of [^13^C] label via non pulmonary methods—loss (excretion). (4) Re‐entry of [^13^C] label from body pool with following elimination—turnover (modified from Sanaka and Nakada (2010))

Various literature define retention and fixation differently and many authors characterize fixation and retention as the same term interchangeably (Sanaka & Nakada, 2010). Fixation and retention can result in a delay in the pulmonary [^13^C]CO_2_ elimination, where some of [^13^C] label is recycled back from the bicarbonate pools to pulmonary circulation and exhaled as [^13^C]CO_2_ (Sanaka & Nakada, 2010). By defining these four processes, important kinetic factors which cause discrepancies between the inlet [^13^C] label contained in the test meal and the outlet [^13^C]CO_2_ exhaled in the breath are shown in Figure 3 (Sanaka & Nakada, 2010). As briefly described earlier, there is a much higher level of complexity in the kinetics of the ^13^C label partially based on bicarbonate kinetics which will not be covered in this theoretical background. However, the work on compartmental modeling of bicarbonate by others supports the development of a more physiological modeling of breath tests (Raj et al., 2006; Saccomani et al., 1991).

## PHARMACOKINETIC MODELING OF GE

There is a large body of literature surrounding physiologically based pharmacokinetic (PBPK) models of GE, with most studies and modeling approaches focusing on the digestion and absorption of drugs and nutrients. However, there are a relatively small number of studies that have attempted to use these models for GE and [^13^C]‐OABT. The aim of these semi‐mechanistic PK models is to derive parameters that have physiologically relevant counterparts, that are more easily identifiable, can accurately model the observed data, and can be used to diagnose and test treatments for GE problems. Such a model could also enable a more satisfactory correlation between indirect ([^13^C]‐OABT) and direct (scintigraphy) measurement of GE.

The approach with PBPK models is to divide the human body into several compartments, which can then be modeled as a set of chemical reactors. Typically, to model digestion, the stomach is considered a continuously stirred tank reactor (CSTR), and usually represented as a first‐order ordinary differential equations (ODE), with the small intestine modeled as a plug flow reactor (PFR), modeled using partial differential equations (PDE). Since these models are usually to simulate the dosage of nutrients or drugs and their absorption, they have tended to simplify many issues that are important in digestion, such as meal properties and interactions and feedback loops within the body (Karatza & Karalis, 2020). Effects such as meal viscosity (Marciani et al., 2001), and feedback mechanisms that respond to meal compositions (Brener et al., 1983), have long been known, but have not been rigorously studied within [^13^C]‐OABT results. Recently, advances in PBPK models for describing many of these previously overlooked mechanisms may provide a mode tailored modeling approach (Moxon et al., 2016, 2017; Yu, 2018). While these models may require many more parameters, many of which may be difficult to estimate, using such a model would allow for more predictive capabilities and allow for more accurate determination of the true gastric emptying times from [^13^C]‐OABT, without the requirement of data/system‐specific corrections that are required from using the non‐compartmental models. In addition to PBPK models, computational fluid dynamics (CFD) models have also been used to study the gastric emptying process (Ishida et al., 2019). However, due to the excessive computational requirements and the difficulty in fitting parameters, these models are unsuitable for clinical use, and we focus on PBPK approaches in this review.

Past research (Ogungbenro & Aarons, 2011) presented work which focused on structural identifiability analysis (SIA) using the Differential Algebra for Identifiability of Systems (DAISY) software package. The aim of their approach was to develop a new semi‐mechanistic model to analyze [^13^C]‐OABT results and investigate the effectiveness of DAISY as a semi‐mechanistic simulation model. The model was designed to classify mathematical model parameters into globally identifiable, locally identifiable, or non‐identifiable in the context of GE, absorption, distribution, metabolism, and elimination processes for [^13^C]octanoic acid.

The authors built upon their previous results and developed a semi‐mechanistic model in MATLAB that went beyond their initial work (Ogungbenro & Aarons, 2011) by analyzing the results of the semi‐mechanistic PK model against results yielded by other known [^13^C]‐OABT methods: Modified exponential method (Siegel et al., 1988), Ghoos model (Ghoos et al., 1993) and Wagner–Nelson approach (Sanaka et al., 2005).

**FIGURE 4:**
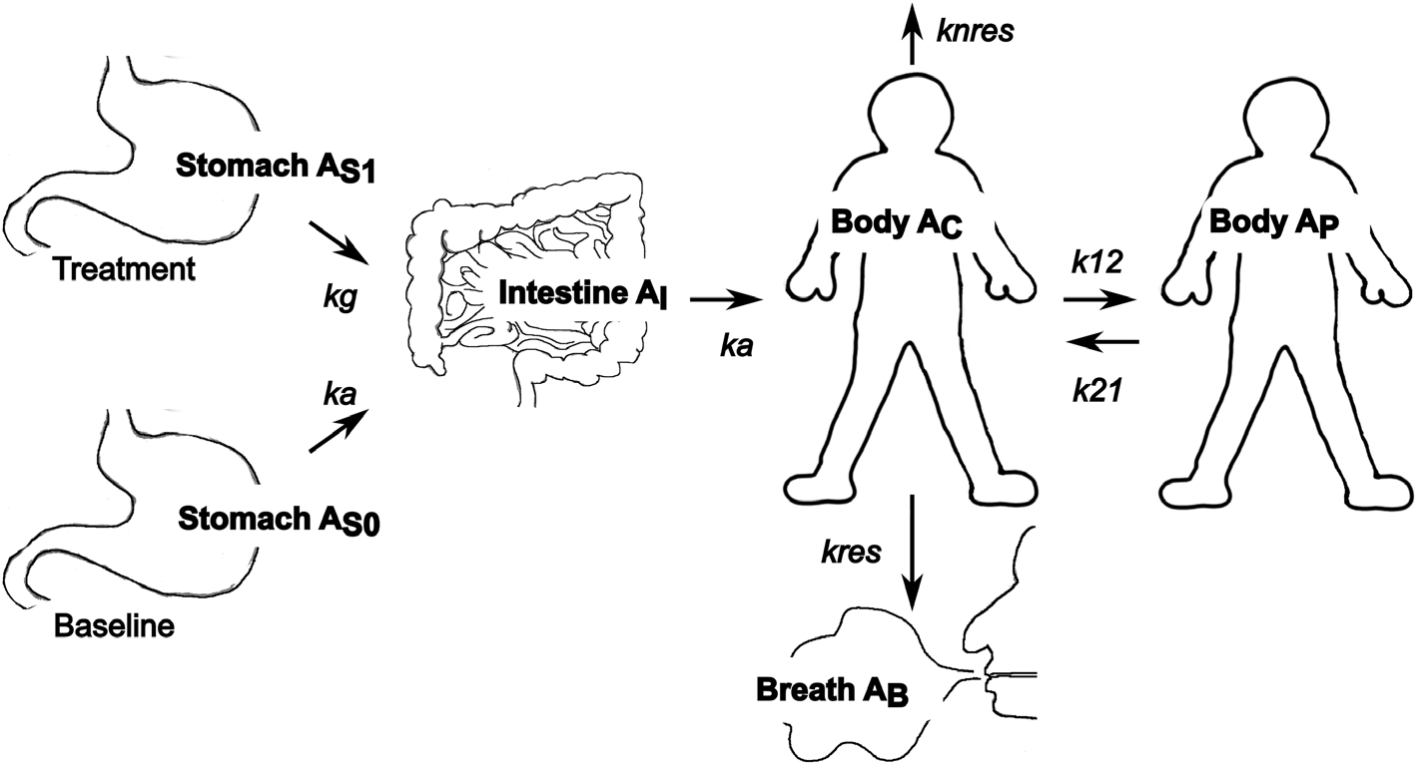
Graphical representation for the semi‐mechanistic model based on five compartments. The five compartments are stomach, small intestine, central and peripheral body, and breath excretion. In this case, showing a treatment and baseline approach, based on data in Ogungbenro and Aarons (2012)

Rate constants representing kinetics of the processes recognized in previous research (Sanaka & Nakada, 2010) were included, such as absorption (with baseline GE as a constraint), expulsion via breath, expulsion via non‐breath routes, central body to peripheral body and peripheral body to central body (representing distribution and metabolism). This is represented in Figure 4 where the input for the model is the stomach with the output in the breath expulsion. It was found in Ogungbenro et al. (Ogungbenro & Aarons, 2011) that a 4‐compartmental model (stomach, small intestine, central body and peripheral body) produced a more effective mode than a 3‐compartmental model, but, in Ogungbenro and Aarons (2012), it was then further found that the 5‐compartmental model (stomach, small intestine, central body, peripheral body and breath) proved to generate a superior model in terms of fit, however it is clear that the large numbers of parameters of these models in relation to the fairly small single‐response datasets may result in overfitting.

Equation (15) through to Equation (19) represent the mass transfer from each compartment within the model, based on rate constants (as presented in Figure 4), where *A*_s_, *A*_I_, *A*_C_, *A*_P_, and *A*_B_ represent the amount of [^13^C] within the stomach, intestines, central body, peripheral body and breath, respectively (Ogungbenro & Aarons, 2012). The transfer rates are based on first order ODEs.

(15) $$\frac{dA_{s}}{\mathrm{dt}}=-\mathrm{kg}∙A_{s}$$

(16) $$\frac{dA_{1}}{\mathrm{dt}}=\left(\mathrm{kg}∙A_{s}\right)-\left(\mathrm{ka}∙A_{I}\right)$$

(17) $$\frac{dA_{c}}{\mathrm{dt}}=\left(\mathrm{ka}∙A_{1}\right)+\left(k21∙A_{P}\right)-\left(k12+\text{kres}+\text{knres}\right)A_{c}$$

(18) $$\frac{dA_{P}}{\mathrm{dt}}=\left(k12∙A_{C}\right)-\left(k21∙A_{P}\right)$$

(19) $$\frac{dA_{b}}{\mathrm{dt}}=\text{kres}∙A_{C}\;$$

This approach proved highly promising, with exceptionally good fits, and which produced favorable results with the best correlation relative to scintigraphic data compared to the other empirical [^13^C]‐OABT methods. The correlation for the kg model against the scintigraphic equivalent was *R* ^2^ = 1 compared to the modified exponential method and Ghoos‐method, both with *R* ^2^ = 0.72, and the Wagner‐Nelson method with *R* ^2^ = 0.79 (Ogungbenro & Aarons, 2012). In the model, all parameters are assumed constant, apart from kg and ka. Absorption, distribution, metabolism, and elimination by means other than breath exhalation is shown to contribute to the discrepancies between [^13^C]‐OABT and scintigraphy data for other models. Discrepancies in the other models are due to invalidation of the [^13^C]‐OABT fundamental assumption where half of [^13^C] recovered in breath is not equivalent to half of [^13^C] emptied from the stomach due to other processes occurring. The authors suggested that these models may find applicability in population studies via mixed‐effects modeling, but also reported certain issues with identifiability of parameters that could be overcome with one additional breath test for each subject (Ogungbenro & Aarons, 2011). A limitation is that the extremely precise representation of scintigraphy data suggests overfitting, as it is unlikely that scintigraphy data is captured so accurately. This approach, with its relatively simple rate expressions also results in parameters that have no direct physical/biological significance. Furthermore, the model assumed a first order rate constant for GE and all other relevant processes. This is generally only applicable to estimating GE rate for liquids and must be further modified for solids by implementing a second order or higher model.

To demonstrate the model on PK applications, the same research group (Ogungbenro et al., 2015) extended the model by focusing on the double‐peaked profile phenomenon due to GE, based on the application of a gastric‐modifying drug known as Levodopa. Many related approaches have been explored in the literature, focusing on drug and nutrient distributions in plasma (Bermejo et al., 2020; Hens & Bolger, 2019; Karatza & Karalis, 2020).

These approaches are now commonplace in pharmaceutical and food sciences applications but have yet to be employed in the [^13^C]‐OABT clinical settings or in nutritional studies. Through consideration of the model structure, as well as the experimental procedure, experiments and models may be co‐designed to ensure that models are relevant, informative, and identifiable for clinical settings. Additionally, modern advances in modeling and understanding the feedback mechanisms and effect of meal type on GE, may help in diagnosis and treatment. Since many nutritional studies have larger datasets and longitudinal designs, many identifiability issues may be overcome. In addition, recent advances in statistical modeling techniques, using artificial intelligence and machine learning, have yet to be applied to [^13^C]‐OABT data, however, there is a recent interest in using these tools together with Electrogastrograms and other medical imagery techniques in measuring GE (Min et al., 2019). Work from Buck et al. (Bluck et al., 2010) used Bayesian hierarchical estimation and showed that more advanced statistical techniques can be used to obtain better parameter estimates, particularly for cases with very delayed GE, or when conventional parameter estimation had failed. Although this was used in conjunction with standard empirical models from Schommartz et al. (1998), the approach shows that more advanced statistical approaches may be of use in many cases.

## CONCLUSIONS AND RECOMMENDATIONS FOR FUTURE WORK

The [^13^C]‐OABT is an inexpensive and accessible test to measure GE and is now being widely deployed in clinical and some research settings. The widespread use of the test can allow for a wide range of gastric conditions to be diagnosed and monitored. In the research field, it is important to use consistent testing methods but there is leeway to develop test meals according to the research question. However, the test meal must adhere to the basic principles of using a validated protocol to incorporate the label into the solid phase of the test meal. The analysis of the isotopic enrichment in breath is straightforward and the establishment of research centres of excellence for collaboration at non‐commercial rates would help to enable good quality research using the [^13^C]‐OABT. In a wider context, modeling the fate of a [^13^C] label in other ingested saturated fatty acids, such as palmitic acid would further our understanding of the mechanistic basis of the metabolic effects of dietary fat.

In addition to these areas with respect to experimental standards and testing, the research community has largely relied on several simple modeling approaches based on analytical solutions to first‐order ODEs that can hinder the understanding of the GE system. With significant advances in mechanistic PBPK modeling approaches in the pharmaceutical and food sciences, as well as the rise of data‐centric statistical machine‐learning approaches for diagnosis and model predictions, there is a significant opportunity to leverage this to derive physically significant parameters and allow for better classification and diagnoses from the [^13^C]‐OABT datasets.

To achieve this, standardization in testing to ensure well‐labeled data sets, with consistent testing methods (meals, activity, etc.) will be required, in addition to the computational modeling challenges. To obtain more physiologically relevant models, combining the [^13^C]‐OABT with other multi‐response experiments will be required to determine kinetics of other elimination pathways within PBPK‐derived approaches, leveraging the experiences of this research community. A further open challenge is in using existing PBPK models to determine the effects of meal composition and drugs on GE and combine this knowledge with the [^13^C]‐OABT.

## AUTHOR CONTRIBUTIONS

Michael Short, Barbara A. Fielding, and Johanna von Gerichten wrote the manuscript. Joe E. Prollins and Marwan H. Elnesr performed basic literature research as part of their MEng. Ishanki A. De Mel, Alan Flanagan, and Jonathan D. Johnston involved in manuscript writing and editing.

## CONFLICT OF INTEREST

The authors declare that they have no conflict of interest and the conclusions of this review are presented clearly, honestly, and without fabrication.

## ETHICAL STATEMENT

This review article does not present studies involving humans and/or animals done by the authors and therefore does not need ethical approval.
